# Characteristic of Maxillofacial Injuries Resulting from Interpersonal Violence Between 2021 and 2025: Retrospective Study from Poznan, Poland

**DOI:** 10.3390/jcm15072556

**Published:** 2026-03-27

**Authors:** Maciej Okła, Szymon Rzepczyk, Jakub Majewski, Maria Szczepaniak, Jakub Jankowski, Czesław Żaba, Kacper Nijakowski

**Affiliations:** 1 Department of Maxillofacial Surgery, Poznan University of Medical Sciences, 60-355 Poznan, Poland; 2 Department of Forensic Medicine, Poznan University of Medical Sciences, 60-806 Poznan, Poland; 3 The Student Scientific Society, Poznan University of Medical Sciences, 60-806 Poznan, Poland; 4 Department of Conservative Dentistry, Poznan University of Medical Sciences, 60-812 Poznan, Poland; 5 Doctoral School, Poznan University of Medical Sciences, 60-812 Poznan, Poland

**Keywords:** facial trauma, maxillofacial injury, facial fracture, interpersonal violence

## Abstract

**Background**: Interpersonal violence is one of the most common causes of maxillofacial injuries. These injuries can range from minor soft-tissue injuries to serious, life-threatening conditions. This is particularly important when injuries occur in an exposed and vulnerable area of the body, such as the facial area. This study aimed to analyse the types of maxillofacial injuries, assess a profile of a typical victim of violence and determine the circumstances of the injury. **Methods**: A retrospective review was performed on the clinical data of patients managed for maxillofacial trauma resulting from interpersonal violence at the Department of Maxillofacial Surgery, University Clinical Hospital, Poznan, spanning the period from 2021 to 2025. **Results**: The study group included 510 patients, of which 95.41% were males, and the median age in the study group was 34 years. Furthermore, 14.71% of patients were under the influence of alcohol at the time of the violent incident. Most injuries occurred in 2022 (25.88%). Regarding months, June had the highest reported incidents (10.59%), while Saturday was the most injury-prone day (25.10%). The median days of hospitalisation in the study group was five. The mandible was the most frequently affected area. The most common types of fractures were single mandible fractures (30.59%) and double mandible fractures (27.25%). Most injuries were treated surgically (96.67%). In 10.20% of cases, the intervention of other specialists was needed. **Conclusions**: It is important to effectively prepare medical staff to receive patients with a history of interpersonal violence to diagnose and treat these types of injuries properly.

## 1. Introduction

Interpersonal violence, especially physical violence, is a significant problem from the point of view of healthcare systems and social policy [1,2,3]. Depending on the circumstances of the incident, it can be categorised as urban violence (UV), intimate partner violence (IPV), domestic violence (DV) and child or elderly abuse [4,5,6,7,8,9]. Cases of violence against females are also significant in the context of maxillofacial injuries [10,11,12]. Its connection with aggression, understood as a set of behaviours aimed at causing harm or injury to another person, is also significant and may translate into somatic findings detected in the examination [13]. It is postulated that interpersonal violence, apart from road accidents and falls, is one of the main causes of maxillofacial fractures [14,15,16]. Injuries may occur in circumstances of assaults, fights or robberies where the primary goal is to obtain material goods. Additionally, not only fists can be used for an attack but also dangerous objects such as batons, brass knuckles or elements of the immediate surroundings, such as branches or glass bottles [17,18]. Therefore, injuries usually occur as a result of blunt trauma. The injuries themselves can take the form of minor trauma, such as bruises or abrasions, as well as wounds and bone fractures [18]. The influence of alcohol or other psychoactive substances is also significant.

A study conducted in Poznan, covering reports from medical–forensic examinations of interpersonal violence cases from 2015 to 2020, showed that the head is the most common location for injuries. These injuries are most often caused by punching, grappling or kicking [19]. Another study conducted in Poland showed that interpersonal violence may constitute over 20% of open reduction internal fixation performed under general anaesthesia in maxillofacial surgery departments [20]. Moreover, the effects of fights or assaults are a common reason for patients reporting to Emergency Departments (EDs) [21,22]. A study conducted in the US showed that head trauma resulting from intentional injury was mainly caused by assaults among patients visiting EDs [23]. Major reasons for aggressive behaviour were altercations and robberies, and the incidents usually involved young males [23]. The face is an exposed body area particularly susceptible to injury [24,25]. Due to the presence of the organs of the sense of smell, vision and hearing in the craniofacial region, as well as the initial sections of the digestive and respiratory systems, the effects of beatings may pose a threat to life or result in permanent disability or aesthetic effects in the form of permanent disfigurement or deformation. This also has a significant impact on the victim’s quality of life, affecting their self-esteem and other aspects of mental health [26,27,28].

This study aimed to determine a typical profile of maxillofacial injuries resulting from physical interpersonal violence, along with an assessment of the circumstances surrounding the events.

## 2. Materials and Methods

Medical records of patients of the Department of Maxillofacial Surgery at the University Hospital of the Poznan University of Medical Sciences over a 5-year period, from the beginning of January 2021 to the end of December 2025, were subjected to a retrospective analysis. The criterion for inclusion in the study group was the presence of information in the medical records indicating the development of maxillofacial trauma as a result of interpersonal violence. Cases identified in the documentation as “unspecified injury” without any event characteristics were excluded from the study.

The cohort of cases fulfilling the predefined inclusion criteria underwent a comprehensive evaluation, focusing on variables such as demographic aspects like gender and age, as well as the patient’s country of origin. Furthermore, the analysis extended to the documented consumption of alcohol or alternative psychoactive agents during the incident, the specific timing and context of the incident, and a detailed assessment of the sustained injuries, with a primary focus on anatomical distribution and the requirement for multidisciplinary specialist consultations. Moreover, comprehensive data on the duration of inpatient stay, the decision on surgical treatment, and the need for multidisciplinary specialist consultations were systematically extracted. This information was subsequently retrieved from the electronic medical record database by two independent investigators and organised into a standardised spreadsheet, facilitating rigorous data processing and formal statistical evaluation. The specific therapeutic modalities employed were not analysed at this stage. The method of collecting and cataloguing data on injuries was modelled and analogous to previously published articles on the subject [29].

The present research paper constitutes a further development of our prior studies into the nature of maxillofacial trauma, focusing on a comprehensive analysis of both the causes and the specific environmental factors involved [30].

**Statistical analysis**

Qualitative variables were compared using Pearson’s Chi-squared test, and quantitative variables were determined using the Mann–Whitney test (due to non-compliance with the normal distribution according to the Shapiro–Wilk test). Continuous data were presented as medians and quartile ranges. Multidimensional correspondence analysis was used to assess the relationships between the most frequent fractures and individual factors, such as gender, age, and alcohol intake. Based on the scree plot, the three-dimensional analysis was chosen as the best description of this association. The significance level was set at α = 0.05 for all analyses. The statistical analysis was performed using Statistica 13.3 (StatSoft, Cracow, Poland).

## 3. Results

Five hundred ten patients, 25 females and 485 males, were qualified for the study group. The median age of participants was 34, and the median number of hospitalisation days was five. Most injury victims were Polish. Only 14.71% of patients had alcohol in their blood. Table 1 presents the detailed demographic data.

Table 2 summarises the frequency of injuries by year, month, and day of the week. Most injuries occurred in 2022 (25.88%). Regarding months, June had the highest reported incidents (10.59%), while Saturday was the most injury-prone day (25.10%).

Table 3 provides a detailed breakdown of the injuries. The mandible was the most frequently affected area. The most common types of fractures were mandible single fractures (30.59%) and double mandible double fractures (27.25%), whereas LeFort 3 fractures were the least reported (0.98%). Fractures more often involved the left side of the patient (44.70%). The tool used to inflict the injury, as well as the reason for injury, was predominantly classified as undefined (95.49% and 72.35%, respectively). Most injuries were treated surgically (96.67%).

Comparisons according to gender, age, and alcohol consumption are presented in Table 4. The comparison of fracture types between genders showed no statistically significant differences across any fracture category, although mandibular fractures were the most frequent in both males and females. When stratified by age, several significant differences emerged. Patients aged ≤30 years had a higher proportion of orbital fractures (20.6% vs. 11.0%, *p*-value = 0.004), LeFort I fractures (3.65% vs. 0.48%, *p*-value = 0.020), LeFort II fractures (4.32% vs. 0.96%, *p*-value = 0.027), and fractures categorised as “others” (2.87% vs. 0.66%, *p*-value = 0.049) compared with patients older than 30 years. In contrast, no significant age-related differences were observed for ZMO, zygomatic, nasal, maxillary, LeFort III, or mandibular fractures.

Alcohol involvement was associated with differences in fracture patterns. Patients with documented alcohol use had a significantly lower proportion of orbital fractures (8.0% vs. 18.16%, *p*-value = 0.029) but a higher proportion of complex mandibular fractures (≥3 fractures) compared with non-alcohol users (8.0% vs. 2.76%, *p*-value = 0.023). No significant differences related to alcohol were observed for other fracture types. Fracture location (left, right, or bilateral) did not differ significantly by gender, age, or alcohol status, with bilateral fractures being the most common overall.

The recorded tool used to inflict the injury was predominantly classified as “undefined” across all groups (>94%), with no significant differences by gender, age, or alcohol use. However, the documented reason for injury differed significantly by gender (*p*-value < 0.001). Intimate partner violence (IPV) was reported in 40% of female patients compared with 0.21% of male patients, whereas assault- and fight-related injuries were more commonly recorded among males. No statistically significant differences in injury reason were observed with respect to age or alcohol use, although assault tended to be more frequent in older patients and in those with alcohol involvement.

Regarding treatment, the majority of patients underwent operative management, with no significant differences by gender or age. However, patients with alcohol involvement were less likely to receive operative treatment (92.0% vs. 97.47%, *p*-value = 0.015) and more likely to be treated on an ambulatory basis. Additionally, alcohol use was associated with a significantly higher rate of additional consultations (17.33% vs. 8.97%, *p*-value = 0.027).

Any differences in hospitalisation days were not observed between males and females. Hospitalisation duration was longer among patients over 30 years old compared to those aged ≤30 years (*p*-value = 0.046). Individuals who consumed alcohol also required longer hospitalisation than those who did not, although this difference was not statistically significant, as shown in Table 5.

**Figure 1:**
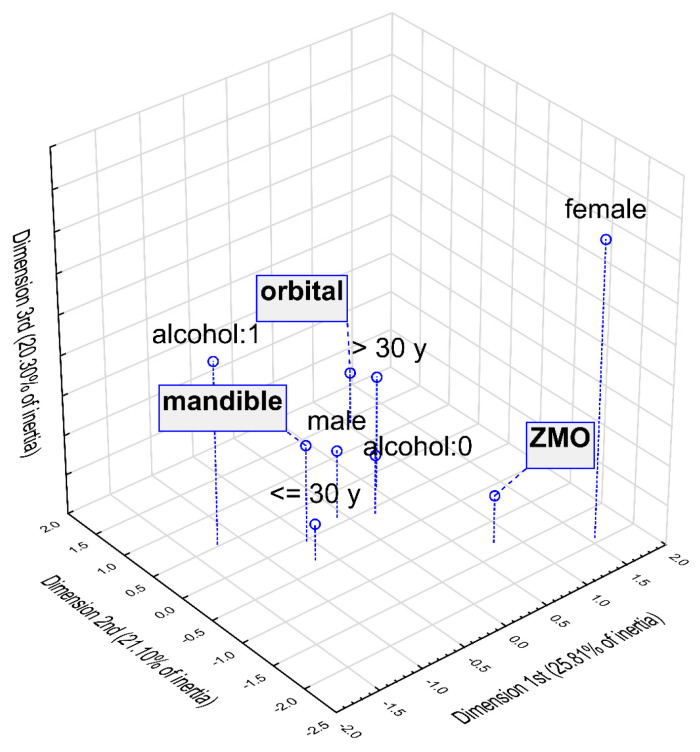
Multidimensional correspondence analysis—3-dimensional plot.

**Figure 2:**
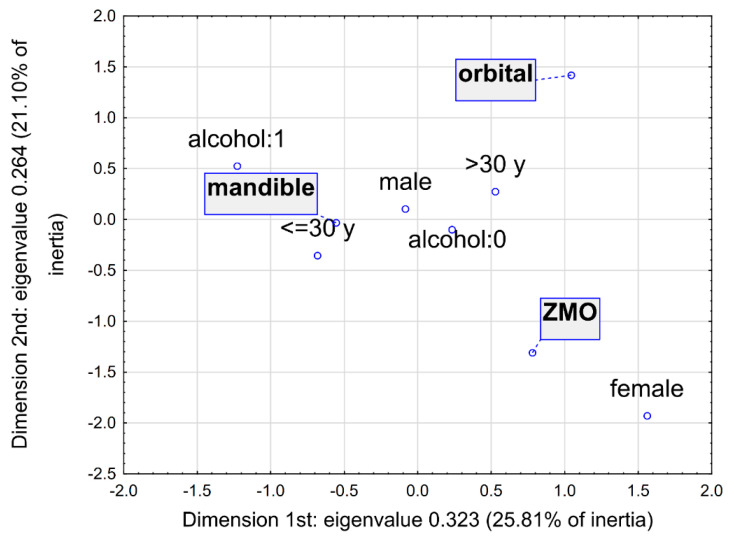
Multidimensional correspondence analysis—2-dimensional plot with the highest inertias.

Multidimensional correspondence analysis was conducted to graphically depict the relationships between the most frequent fractures and individual factors, such as gender, age, and alcohol intake. Figure 1 and Figure 2 show the results of this analysis in three dimensions and in the two dimensions selected with the highest inertias. Parameters characterising the determined points are presented in Table 6. Based on the three-dimensional plot, points representing male gender, age below 30 years, and alcohol intake are clustered closest to the mandible, indicating a higher probability of mandibular fractures in these patients. Due to the smallest angle with the vertex at the beginning of the coordinate system, this relationship is confirmed by the two-dimensional plot. Also, the two-dimensional analyses indicated a relationship between increased incidence of orbital fractures with age over 30 years and ZMO fractures with female gender.

## 4. Discussion

Among the cases included in the study group, namely requiring professional medical assistance after maxillo-facial trauma due to interpersonal violence, injuries occur more frequently in males. Furthermore, typical victims of maxillofacial trauma due to interpersonal violence are young adults. This is consistent with data available in the literature; an analysis conducted in Brazil found that young adult males were more likely to sustain serious maxillofacial injuries as a result of assaults compared to the female population [31]. Regarding medical treatment, males in the study group required longer hospitalisation than females. This indicates that males may suffer more severe injuries as a result of violence. Moreover, in cases of maxillofacial fractures resulting from mechanical injury, the therapeutic method used is usually operative treatment such as a stable miniplate steosynthesis, which should be performed within 72 h of the event. Additionally, another study from Brazil showed that males are more likely to sustain maxillofacial injuries as a result of urban violence and females as a result of domestic violence [29]. In the analysed group, 40% of females reported IPV as the cause of injury. However, in some cases, the circumstances of violence remain unknown based on medical data. This phenomenon may lead to an underestimation of the number of maxillofacial injuries resulting from domestic violence in females. The same problem of insufficient data regarding the violent incident in medical documentation and underestimation of domestic violence resulting in maxillofacial injuries in females was reported in the literature [32]. Most of the victims were Polish, but patients of post-Soviet nationality accounted for 10.20% of all cases. This can be explained as an influx of people from countries along Poland’s eastern border due to the geo-military situation. Particularly significant in this aspect is the issue of emigration from Ukraine since the beginning of the armed conflicts taking place in its territory. Regarding violent behaviours in this group, possible challenges with living in a new country, such as social and economic difficulties related to migration, should not be underestimated. Issues related to the language barrier and cultural background, which may constitute a risk factor for violence, are also significant [26]. In this context, maxillofacial injuries resulting from military operations also become important [33]. The percentage of foreigners in the study group is lower than that in some studies in the literature; the analysis conducted in Turin showed that foreigners constituted over 34% of patients, with most of them from ex-Soviet-aligned countries and Africa [34]. Moreover, research indicates that assaults are a key aetiology of maxillofacial trauma among foreigners [35].

In the analysed period, the number of cases in 2021 was the lowest, while in 2022, it was the highest. This may be related to the COVID-19 pandemic situation, especially in the form of lockdowns, home isolation, and some sanitary restrictions still in force in 2021. However, in 2022, with many social restrictions loosened and mass events returning, the number of incidents increased. Scholarly reports offer varied data regarding the prevalence of violence-related injuries during the pandemic. Analyses conducted in the Netherlands have shown a reduction in the number of maxillofacial injuries, including those resulting from violence during lockdowns [36]. A similar situation was observed in New Zealand, where the number of hospital admissions due to maxillofacial injuries as a result of interpersonal violence decreased during lockdowns [37]. However, evidence from research conducted in the United States indicates an uptick in the number of maxillofacial fractures resulting from interpersonal assaults during the implementation of social distancing protocols [38]. Research from Italy showed that the number of domestic traumas increased, especially among females and children during lockdowns [39]. Studies from various countries, as well as multinational meta-analyses, show that an increase in the number of domestic violence incidents during pandemics was also observed in other countries [40,41,42,43]. Changes in the profile of high-energy injuries in the maxillofacial region were also observed in Poland [44]. Moreover, attention is drawn to the possible underreporting of such events during lockdowns [45].

Most cases occurred during warm months and on weekends, especially on Saturday. This may be related to higher alcohol consumption and more frequent organisation of mass events in months with good weather conditions and weekends. Some of the academic literature consistently highlights a positive correlation between the incidence of maxillofacial trauma and weekend periods. This trend is frequently attributed to lifestyle factors and specific socio-recreational activities prevalent during these intervals, such as drinking alcohol or partying [46,47]. Moreover, in the study population, over 14% of patients were under the influence of alcohol at the time of the incident. In a New Zealand study, drugs, alcohol, or both were found in over 37% of victims of interpersonal violence who sustained facial fractures [48].

After assessing injury profiles in terms of gender, single mandible fractures were the most frequent in males, while in females, double mandible fractures were the most common. The observed distribution of injuries reflects the trends established in the multicentre research, which remains one of the most comprehensive prospective studies on European maxillofacial trauma. Analysis showed that in cases of interpersonal violence, the mandible is the primary site of fracture due to its prominent position and the nature of blunt force impact. Furthermore, the study highlighted a high incidence of middle-face trauma, specifically involving the orbito–zygomatic–maxillary complex and the orbital structures [49]. Moreover, studies focusing on maxillofacial injuries in females who are victims of domestic violence indicate that in cases of domestic violence, the maxilla–zygomatic complex and mandible are more frequently fractured [50]. Research conducted in the USA suggests a higher incidence of orbital fractures in this population [51]. Additionally, studies on the facial injury profile in cases of IPV have shown that the victims are mainly females, and fractures usually occur in the nasal bones, mandible, and orbits [52]. Due to their potential consequences (e.g., enophthalmos), orbital fractures also become particularly important [53]. Orbital fractures appeared to be slightly more common among female patients, which can be explained by a less massive structure and the thinner, sharper orbital rims characteristic of the female skull, resulting in decreased resistance to mechanical forces. Regarding the age-related difference in the proportion of orbital fractures in patients aged over 30 years, a potential explanation may be differences in bone elasticity and periosteal thickness, as well as the age-associated reduction in bone strength. This phenomenon is primarily driven by the progressive degradation of the bone microarchitecture, which contributes to increased skeletal fragility within the orbital region.

Considering assaults as a cause of maxillofacial injuries, a study conducted in the UK showed that fractures most frequently occur in the mandible, followed by the orbit and zygoma [54]. Similar observations were made in a study conducted in Italy, which characterised interpersonal violence-related facial fractures in southern Europe, further indicating that approximately 14% of patients suffered multiple fractures [55]. A study conducted in Poland has also shown that in cases of assaults, fractures most often occur in the lower face area, the victims are usually young males, and the incidents occur more often in urban areas [56]. A significant portion of double mandibular fractures stems from the intricate biomechanics of the mandible. Such injuries often manifest at both the primary impact site and a remote location, frequently the contralateral side, due to the propagation of forces through the bone, which leads to structural failure at its weakest points [57,58,59,60].

Furthermore, a higher prevalence of fractures was observed on the left side of the patient’s facial area, a clinical finding that potentially correlates with the right-hand dominance of the assailants. This asymmetry is consistent with the established literature regarding interpersonal violence, particularly in domestic contexts, where left-sided traumas are documented with greater frequency than right-sided injuries [61,62]. This phenomenon may be attributed to the positioning during face-to-face confrontations, where a right-handed perpetrator’s strike lands on the victim’s left side. Consequently, the lateralization of these injuries may serve as a diagnostic indicator for medical professionals and forensic experts when reconstructing the mechanics of an assault and identifying patterns of intentional physical abuse.

It is also imperative to acknowledge that the patient cohort detailed in this study is specifically limited to individuals whose injuries necessitated inpatient hospitalisation and specialised care within a maxillofacial surgery department. This distinction is important, as the current clinical literature indicates that patients presenting with facial skeleton fractures resulting from domestic violence are observed in approximately 30–50% of victims [18,63]. The vast majority of individuals affected by such incidents sustain relatively minor trauma that does not require surgical intervention. These more frequent injuries typically involve soft-tissue damage, including bruising, extensive lacerations, contusions, and cutaneous abrasions, which are often managed in outpatient settings [24,64,65,66]. Consequently, the data presented here reflects the more severe end of the trauma spectrum rather than the total epidemiology of violence-related injuries. Beyond immediate surgical intervention, victims of maxillofacial violence often necessitate multidisciplinary outpatient care. Such trauma can induce acute masticatory dysfunction, including Temporomandibular Disorders (TMDs). Given that symptoms may manifest later, implementing highly sensitive TMD screening during every follow-up consultation is essential for effective post-traumatic management [67].

Research limitations include missing data on event specifics, such as the injury mechanism and its circumstances, in certain medical files. Furthermore, specific instances might be omitted from this analysis as details of physical altercations or assaults were frequently undocumented in clinical notes. This occurs especially among individuals relocated from different medical centres and those admitted from ambulatory services, with the latter providing details significantly after the occurrence. A comparable issue exists in identifying the precise proportion of individuals intoxicated by alcohol or narcotics during the trauma, since these screenings are not standard practice. Victims might present at the facility long after the violent episode, resulting in an incorrectly reduced frequency of subjects recorded as being under the influence of mind-altering substances when the original incident took place. Regarding the tool used to inflict the injuries, victims are often unaware or unsure of what the perpetrator used to inflict the injuries, so in many cases, this precise information was missing.

## 5. Conclusions

The phenomenon of interpersonal violence in physical terms is associated with significant health consequences in the form of maxillofacial injuries. The analysis performed showed that the victims were usually men, and the fractures occurred in the mandible. Furthermore, incidents of violence resulting in maxillofacial fractures usually occur during warm months and weekends. In the vast majority of cases, surgical treatment is necessary, and fractures occurred more often on the left side. Cases of maxillofacial fractures resulting from intimate partner violence were more common in women. Identifying high-risk groups allows for the implementation of preventive measures. This is particularly important in the context of the percentage of females sustaining maxillofacial injuries due to domestic violence, despite constituting a small percentage in the analysed group.

Further research is needed to identify other typical circumstances in which these incidents occur. This will enable coordinated action by healthcare providers and authorities. Additionally, it is important to properly prepare medical staff for receiving patients with a history of interpersonal violence. Beyond routine diagnostics for trauma patients, it is worth extending examinations to include consultations in maxillofacial surgery. Special attention should be paid to the occurrence of mandibular fractures. This is crucial due to potential complications, such as permanent deficits in mastication and speech functions, as well as aesthetic deformities of the face. In such cases, prompt surgical treatment is essential.

## Figures and Tables

**Table 1:** Participants’ characteristics.

	M	Q1–Q3
**Age, years**	34	26–44
**Hospitalisation, days**	5	4–7
	*n*	%
**Gender**		
Males	485	95.10
Females	25	4.90
**Nationality**		
Polish	452	88.63
Post-Soviet	52	10.20
Others	6	1.17
**Alcohol**	75	14.71

**Table 2:** Frequency of injuries depending on year, month, and day of the week.

	*n*	%
**Year**		
2021	85	16.67
2022	132	25.88
2023	93	18.23
2024	87	17.06
2025	113	22.16
**Month**		
I	33	6.47
II	34	6.67
III	36	7.06
IV	43	8.43
V	51	10.00
VI	54	10.59
VII	51	10.00
VIII	52	10.20
IX	44	8.63
X	34	6.67
XI	39	7.64
XII	39	7.64
**Day**		
Monday	45	8.82
Tuesday	49	9.61
Wednesday	55	10.79
Thursday	50	9.80
Friday	66	12.94
Saturday	128	25.10
Sunday	117	22.94

**Table 3:** Detailed characteristics of injuries.

	*n*	%
**Fracture kind**		
ZMO	86	16.86
Orbital	85	16.67
Zygomatic	21	4.12
Nasal	28	5.49
Maxilla	19	3.73
LeFort1	12	2.35
LeFort2	15	2.94
LeFort3	5	0.98
Mandible = 1	156	30.59
Mandible = 2	139	27.25
Mandible >= 3	18	3.53
Others	8	1.57
**Fracture site**		
Left	228	44.70
Right	113	22.16
Both	169	33.14
**Tool used**		
Undefined	487	95.49
Fist	11	2.16
Dull	12	2.35
**Injury reason**		
Undefined	369	72.35
IPV	11	2.16
Assault	106	20.78
Robbery	5	0.98
Fight	19	3.73
**Treatment method**		
Operative	493	96.67
Ambulatory	17	3.33
Other consultations	52	10.20

**Table 4:** Comparison of injury characteristics depending on gender, age, and alcohol intake.

	Gender			Age			Alcohol		
	Males *n* = 485	Females *n* = 25	*p*-Value	≤30 *n* = 209	>30 *n* = 301	*p*-Value	No *n* = 435	Yes *n* = 75	*p*-Value
Fracture kind									
ZMO	16.49	24.00	0.328	13.40	19.27	0.082	16.55	18.67	0.651
Orbital	16.49	20.00	0.647	11.00	20.60	0.004 *	18.16	8.00	0.029 *
Zygomatic	4.33	0	0.288	4.78	3.65	0.528	4.37	2.67	0.494
Nasal	5.57	4.00	0.737	4.78	5.98	0.560	5.52	5.33	0.949
Maxilla	3.71	4.00	0.941	3.35	3.99	0.709	3.21	6.67	0.145
LeFort1	2.47	0	0.426	0.48	3.65	0.020 *	2.30	2.67	0.846
LeFort2	3.09	0	0.372	0.96	4.32	0.027 *	2.53	5.33	0.184
LeFort3	1.03	0	0.610	0.48	1.33	0.338	0.69	2.67	0.109
Mandible = 1	31.13	20.00	0.239	34.45	27.91	0.115	30.57	30.67	0.987
Mandible = 2	27.01	32.00	0.585	30.62	24.92	0.155	26.67	30.67	0.472
Mandible ≥ 3	3.30	8.00	0.214	2.87	3.99	0.502	2.76	8.00	0.023 *
Others	1.65	0	0.517	2.87	0.66	0.049 *	1.38	2.67	0.407
Fracture site									
Left	44.95	40.00	0.889	43.06	45.85	0.812	44.60	45.33	0.175
Right	22.06	24.00		22.49	21.93		23.45	14.67	
Both	32.99	36.00		34.45	32.22		31.95	40.00	
Tool used									
Undefined	95.46	96.00	0.647	95.69	95.35	0.826	95.63	94.66	0.928
Fist	2.27	0		2.39	1.99		2.07	2.67	
Dull	2.27	4.00		1.92	2.66		2.30	2.67	
Injury reason									
Undefined	73.81	44.00	<0.001 *	77.03	69.10	0.123	74.25	61.33	0.076
IPV	0.21	40.00		1.44	2.66		2.30	1.33	
Assault	21.03	16.00		15.79	24.25		19.08	30.67	
Robbery	1.03	0		0.96	1.00		0.69	2.67	
Fight	3.92	0		4.78	2.99		3.68	4.00	
Treatment method									
Operative	96.70	96.00	0.849	96.17	97.01	0.604	97.47	92.00	0.015 *
Ambulatory	3.30	4.00		3.83	2.99		2.53	8.00	
Other consultations	9.90	16.00	0.325	7.18	12.29	0.060	8.97	17.33	0.027 *

*: statistically significant difference.

**Table 5:** Comparisons of hospitalisation duration depending on gender, age, and alcohol intake.

	Gender			Age			Alcohol		
	Males *n* = 485	Females *n* = 25	*p*-Value	≤30 *n* = 209	>30 *n* = 301	*p*-Value	No *n* = 435	Yes *n* = 75	*p*-Value
Hospitalisation, days	5 (4–7)	5 (3–8)	0.453	5 (4–7)	6 (4–8)	0.046 *	5 (4–7)	6 (4–8)	0.099

*: statistically significant difference.

**Table 6:** Multidimensional correspondence analysis—parameters of determined points.

	x	y	z	Quality	Relative Inertia	x		y		z	
						Inertia	cos^2^	Inertia	cos^2^	Inertia	cos^2^
**male**	−0.084	0.104	−0.144	0.718	0.010	0.005	0.131	0.010	0.200	0.019	0.387
**female**	1.561	−1.930	2.687	0.718	0.190	0.096	0.131	0.180	0.200	0.362	0.387
**≤30 y**	−0.682	−0.354	−0.536	0.679	0.113	0.157	0.360	0.052	0.097	0.123	0.222
**>30 y**	0.528	0.274	0.415	0.679	0.087	0.122	0.360	0.040	0.097	0.095	0.222
**ZMO**	0.781	−1.311	−0.395	0.579	0.162	0.089	0.142	0.308	0.401	0.029	0.036
**mandible**	−0.555	−0.033	0.224	0.591	0.076	0.148	0.506	0.001	0.002	0.031	0.083
**orbital**	1.044	1.418	−0.343	0.750	0.162	0.160	0.254	0.360	0.469	0.022	0.027
**alcohol:0**	0.234	−0.100	−0.248	0.662	0.032	0.036	0.287	0.008	0.053	0.051	0.323
**alcohol:1**	−1.227	0.525	1.302	0.662	0.168	0.187	0.287	0.042	0.053	0.267	0.323

## Data Availability

Data are available upon request from the corresponding author.

## References

[1] Rosenberg M.L., Butchart A., Mercy J., Narasimhan V., Waters H., Marshall M.S. (2006). Interpersonal Violence. Disease Control Priorities in Developing Countries.

[2] Knapp R. (2011). The Impact of Interpersonal Violence on Health Care. Nurs. Clin..

[3] Kwan K., Wiebe D., Cerdá M., Goldman-Mellor S. (2019). Repeat Assault Injury Among Adolescents Utilizing Emergency Care: A Statewide Longitudinal Study. J. Emerg. Med..

[4] De Souza Cantão A.B.C., Da Silva Lima T.C., Fernandes M.I.A.P., Nagendrababu V., Bastos J.V., Levin L. (2024). Prevalence of Dental, Oral, and Maxillofacial Traumatic Injuries among Domestic Violence Victims: A Systematic Review and Meta-analysis. Dent. Traumatol..

[5] Cohn J.E., Smith K.C., Licata J.J., Michael A., Zwillenberg S., Burroughs T., Arosarena O.A. (2020). Comparing Urban Maxillofacial Trauma Patterns to the National Trauma Data Bank©. Ann. Otol. Rhinol. Laryngol..

[6] Bernardino Í.D.M., Da Nóbrega L.M., De Souza L.T., Ribeiro Monteiro de Figueiredo T., Massoni A.C.D.L.T., d’Ávila S. (2024). Spatial–Temporal Distribution of Maxillofacial Injuries Resulting from Intimate Partner Violence against Women. Dent. Traumatol..

[7] Euerle B., Kelly B. (2005). Maxillofacial Injuries: Imaging, Management, and Disposition. Pediatr. Emerg. Med. Rep..

[8] Michalak P., Wyszyńska-Pawelec G., Szuta M., Hajto-Bryk J., Zapała J., Zarzecka J.K. (2021). Fractures of the Craniofacial Skeleton in the Elderly: Retrospective Studies. Int. J. Environ. Res. Public. Health.

[9] Bojino A., Roccia F., Carlaw K., Aquilina P., Rae E., Laverick S., Romeo I., Iocca O., Copelli C., Sobrero F. (2022). A Multicentric Prospective Analysis of Maxillofacial Trauma in the Elderly Population. Dent. Traumatol..

[10] Zeitler D.L. (2007). The Abused Female Oral and Maxillofacial Surgery Patient: Treatment Approaches for Identification and Management. Oral Maxillofac. Surg. Clin. N. Am..

[11] Attyia M.A., Bede S.Y. (2025). Maxillofacial Trauma in Females: A Retrospective Study. J. Craniofac. Surg..

[12] Da Nóbrega L.M., Bernardino Í.D.M., Barbosa K.G.N., E Silva J.A.L., Massoni A.C.D.L.T., d’Avila S. (2017). Pattern of Oral-maxillofacial Trauma from Violence against Women and Its Associated Factors. Dent. Traumatol..

[13] Rzepczyk S., Nijakowski K., Jankowski J., Nowicki F., Żaba C. (2025). Salivary Markers of Violence—The Possible Alterations in Salivary Cortisol Levels to Identify Victims of Violence—A Systematic Review. J. Forensic Leg. Med..

[14] Palmela Pereira C.M., Resende Dos Santos A., Rodrigues Gonçalves C., Nushi V., Coutinho F., e Silva F.J.S., de Sousa Santos R.F.V. (2023). Retrospective Study of Oral and Maxillofacial Trauma in Portuguese Population. Acta Stomatol. Croat..

[15] Goedecke M., Thiem D.G.E., Schneider D., Frerich B., Kämmerer P.W. (2019). Through the Ages—Aetiological Changes in Maxillofacial Trauma. Dent. Traumatol..

[16] Mogajane B., Mabongo M. (2018). Epidemiology of Maxillofacial Fractures at Two Maxillofacial Units in South Africa. S. Afr. Dent. J..

[17] Sousa R., Bernardino I., Castro R., Cavalcanti A., Bento P., d’Ávila S. (2016). Maxillofacial Trauma Resulting from Physical Violence against Older Adults: A4-Year Study in a Brazilian Forensic Service. Pesqui. Bras. Odontopediatr. Clínica Integr..

[18] Le B.T., Dierks E.J., Ueeck B.A., Homer L.D., Potter B.F. (2001). Maxillofacial Injuries Associated with Domestic Violence. J. Oral Maxillofac. Surg..

[19] Rzepczyk S., Dolińska-Kaczmarek K., Burchardt B., Skowrońska D., Hałasiński P., Bielecka A., Koniarek K., Żaba C. (2023). Prevalence of Physical Violence in the Medical-Forensic Approach in the Years 2015–2020 in City and Neighboring Municipalities: Perspectives from Poland—Poznań Study. Int. J. Environ. Res. Public Health.

[20] Michalik W., Toppich J., Łuksza A., Bargiel J., Gąsiorowski K., Marecik T., Szczurowski P., Wyszyńska-Pawelec G., Gontarz M. (2025). Exploring the Correlation of Epidemiological and Clinical Factors with Facial Injury Severity Scores in Maxillofacial Trauma: A Comprehensive Analysis. Front. Oral Health.

[21] Ogendi J., Ayisi J. (2011). Causes of Injuries Resulting in a Visit to the Emergency Department of a Provincial General Hospital, Nyanza, Western Kenya. Afr. Health Sci..

[22] Cunningham R.M., Carter P.M., Ranney M., Zimmerman M.A., Blow F.C., Booth B.M., Goldstick J., Walton M.A. (2015). Violent Reinjury and Mortality Among Youth Seeking Emergency Department Care for Assault-Related Injury: A 2-Year Prospective Cohort Study. JAMA Pediatr..

[23] Gaw C.E., Zonfrillo M.R. (2016). Emergency Department Visits for Head Trauma in the United States. BMC Emerg. Med..

[24] Saddki N., Suhaimi A.A., Daud R. (2010). Maxillofacial Injuries Associated with Intimate Partner Violence in Women. BMC Public Health.

[25] Toruńska E., Engelgardt P., Szwajkowska M., Krzyżanowski M. (2024). Rare Consequences of a Single Fist Punch to the Orbital Region—A Description of Two Cases = Rzadkie Konsekwencje Pojedynczego Uderzenia Pięścią w Okolicę Oczodołu—Opis Dwóch Przypadków. Arch. Forensic Med. Criminol..

[26] Loutroukis T., Loutrouki E., Klukowska-Rötzler J., Koba S., Schlittler F., Schaller B., Exadaktylos A.K., Doulberis M., Srivastava D.S., Papoutsi S. (2020). Violence as the Most Frequent Cause of Oral and Maxillofacial Injuries among the Patients from Low- and Middle-Income Countries—A Retrospective Study at a Level I Trauma University Emergency Department in Switzerland. Int. J. Environ. Res. Public Health.

[27] Mao J., Li X., Cao K., Xue J., Wang M., Yan D., Zhou Z. (2023). Epidemiology of Maxillofacial Fractures in Northwest China: An 11-Year Retrospective Study of 2240 Patients. BMC Oral Health.

[28] Gvazava S., Margvelashvili V., Chikhladze N., Dulf D., Peek-Asa C. (2023). A Retrospective Study of the Maxillofacial Injuries in Two Emergency Departments in Tbilisi, Georgia. Georgian Med. News.

[29] Ferreira M.C., Batista A.M., Ferreira F.D.O., Ramos-Jorge M.L., Marques L.S. (2014). Pattern of Oral–Maxillofacial Trauma Stemming from Interpersonal Physical Violence and Determinant Factors. Dent. Traumatol..

[30] Nijakowski K., Rzepczyk S., Szczepaniak M., Majewski J., Jankowski J., Żaba C., Okła M. (2025). Characteristics of Bicycle-Related Maxillofacial Injuries Between 2019–2023—Retrospective Study from Poznan, Poland. J. Clin. Med..

[31] Maia L.V.A., Bernardino Í.M., Ferreira E.F., d’Ávila S., Martins R.C. (2018). Exposure to Violence, Victimization Differences and Maxillofacial Injuries in a Brazilian State Capital: A Data Mining Approach. J. Public Health.

[32] Huang V., Moore C., Bohrer P., Thaller S.R. (1998). Maxillofacial Injuries in Women. Ann. Plast. Surg..

[33] Prysiazhniuk O., Palyvoda R., Chepurnyi Y., Pavlychuk T., Chernogorskyi D., Fedirko I., Sazanskyi Y., Kalashnikov D., Kopchak A. (2025). War-Related Maxillofacial Injuries in Ukraine: A Retrospective Multicenter Study. Arch. Craniofacial Surg..

[34] Roccia F., Savoini M., Ramieri G., Zavattero E. (2016). An Analysis of 711 Victims of Interpersonal Violence to the Face, Turin, Italy. J. Cranio-Maxillofac. Surg..

[35] Bonavolontà P., Orabona G.D., Abbate V., Vaira L.A., Lo Faro C., Petrocelli M., Attanasi F., De Riu G., Iaconetta G., Califano L. (2017). The Epidemiological Analysis of Maxillofacial Fractures in Italy: The Experience of a Single Tertiary Center with 1720 Patients. J. Cranio-Maxillofac. Surg..

[36] Boom L.J., Wolvius E.B., Rozeboom A.V.J. (2022). Impact of COVID-19 Lockdown on Incidence of Maxillofacial Fractures: A Retrospective Analysis. Adv. Oral Maxillofac. Surg..

[37] Allen-Brough C.A., Jayakar R., Sim D., MacKinnon C.A., Tan S.T. (2024). Effect of COVID-19 Lockdowns on Maxillofacial Fractures in New Zealand: A Retrospective Study. Australas. J. Plast. Surg..

[38] Ludwig D.C., Nelson J.L., Burke A.B., Lang M.S., Dillon J.K. (2021). What Is the Effect of COVID-19-Related Social Distancing on Oral and Maxillofacial Trauma?. J. Oral Maxillofac. Surg..

[39] Ferragina F., Barca I., Sorrentino A., Kallaverja E., Piloni S., Arrotta A., Cristofaro M.G. (2022). Effect of COVID-19 Italian Lockdown on Maxillofacial Trauma Related to Domestic Violence: A Retrospective Cohort Study. Life.

[40] Naran-Ochir O., Narantsetseg T., Bayartsogt B., Batbileg B., Gan-Ochir B., Altannamar M., Batbayar E. (2024). A Cross-sectional Study of the Impact of the COVID-19 Lockdown on Domestic Violence-related Oral and Maxillofacial Injuries. Dent. Traumatol..

[41] McCrary J., Sanga S. (2021). The Impact of the Coronavirus Lockdown on Domestic Violence. Am. Law Econ. Rev..

[42] Kourti A., Stavridou A., Panagouli E., Psaltopoulou T., Spiliopoulou C., Tsolia M., Sergentanis T.N., Tsitsika A. (2023). Domestic Violence During the COVID-19 Pandemic: A Systematic Review. Trauma Violence Abus..

[43] Piquero A.R., Jennings W.G., Jemison E., Kaukinen C., Knaul F.M. (2021). Domestic Violence during the COVID-19 Pandemic—Evidence from a Systematic Review and Meta-Analysis. J. Crim. Justice.

[44] Bień M., Drogoszewska B., Polcyn A., Michcik A., Garbacewicz Ł. (2024). A Comparison of the Specific Facial Trauma Cases at the Department of Maxillofacial Surgery, Gdansk, Poland, from March 2019 to August 2023. J. Clin. Med..

[45] Klimczak J., Ostaszewski P., Włodarczyk-Madejska J. (2023). “There Were Fewer Calls, Even Though There Was More Violence”: Domestic Violence in Poland during the COVID-19 Pandemic. Biul. Kryminol..

[46] Kapoor P., Kalra N. (2012). A Retrospective Analysis of Maxillofacial Injuries in Patients Reporting to a Tertiary Care Hospital in East Delhi. Int. J. Crit. Illn. Inj. Sci..

[47] Gilthorpe M.S., Wilson R.C., Moles D.R., Bedi R. (1999). Variations in Admissions to Hospital for Head Injury and Assault to the Head Part 1: Age and Gender. Br. J. Oral Maxillofac. Surg..

[48] York B., Sent-Doux K., Heo J., Barnett M., Marsh R., Mackinnon C., Tan S. (2019). Interpersonal Violence and Maxillofacial Fractures. Ann. Maxillofac. Surg..

[49] Boffano P., Roccia F., Zavattero E., Dediol E., Uglešić V., Kovačič Ž., Vesnaver A., Konstantinović V.S., Petrović M., Stephens J. (2015). Assault-Related Maxillofacial Injuries: The Results from the European Maxillofacial Trauma (EURMAT) Multicenter and Prospective Collaboration. Oral Surg. Oral Med. Oral Pathol. Oral Radiol..

[50] Levin L., Goldman S., Lin S., Radomislensky I., Savitsky B. (2024). Dental and Maxillofacial Injuries Associated with Domestic Violence against Women in Israel: A Report for 2011–2021. Dent. Traumatol..

[51] Arosarena O.A., Fritsch T.A., Hsueh Y., Aynehchi B., Haug R. (2009). Maxillofacial Injuries and Violence against Women. Arch. Facial Plast. Surg..

[52] Gujrathi R., Tang A., Thomas R., Park H., Gosangi B., Stoklosa H.M., Lewis-O’Connor A., Seltzer S.E., Boland G.W., Rexrode K.M. (2022). Facial Injury Patterns in Victims of Intimate Partner Violence. Emerg. Radiol..

[53] Koryczan P., Zapała J., Gontarz M., Wyszyńska-Pawelec G. (2021). Comparison of the Results of the Treatment of Enophthalmos in Orbital Blowout Fracture in Children/Adolescents and Adults. Dent. Med Probl..

[54] Abukhder M., Mobarak D. (2022). A Retrospective Cohort Study on the Aetiology and Characteristics of Maxillofacial Fractures Presenting to a Tertiary Centre in the UK. Ann. Med. Surg..

[55] Cirignaco G., Catarzi L., Monarchi G., Committeri U., Frosolini A., Togni L., Mascitti M., Balercia P., Santarelli A., Consorti G. (2025). Interpersonal Violence-Related Facial Fractures: 12-Year Trends and Surgical Outcomes in a Southern European Level-I Trauma Centre. Medicina.

[56] Parulska O., Dobrzyński M., Bazan J., Całkosiński I. (2017). Epidemiological Assessment of Maxillofacial Fractures in the Inhabitants of Lower Silesia, Poland in 2002–2006—Pattern of Maxillofacial Fracture. Pol. Ann. Med..

[57] Teresinski G. (2019). Postacie i Mechanizmy Złamań Twarzoczaszki. Medycyna Sądowa.

[58] van Eijden T. (2000). Biomechanics of the Mandible. Crit. Rev. Oral Biol. Med..

[59] Pappachan B., Alexander M. (2012). Biomechanics of Cranio-Maxillofacial Trauma. J. Maxillofac. Oral Surg..

[60] Unnewehr M., Homann C., Schmidt P.F., Sotony P., Fischer G., Brinkmann B., Bajanowski T., DuChesne A. (2003). Fracture Properties of the Human Mandible. Int. J. Leg. Med..

[61] Yamamoto K., Matsusue Y., Horita S., Murakami K., Sugiura T., Kirita T. (2019). Maxillofacial Fractures Associated with Interpersonal Violence. J. Craniofac. Surg..

[62] Eggensperger N., Smolka K., Scheidegger B., Zimmermann H., IIZuka T. (2007). A 3-Year Survey of Assault-Related Maxillofacial Fractures in Central Switzerland. J. Cranio-Maxillofac. Surg..

[63] Yari A., Fasih P., Bagheri A., Aryanezhad S.S., Sani M.K. (2024). Prevalence and Pattern of Maxillofacial Injuries Associated with Domestic Violence: A Retrospective Study at a Major Trauma Center. Dent. Traumatol..

[64] Benassi C.M., De Assis Santos V.P., Spagnol G., Ferraz E.P., Luz J.G.C. (2024). The Profile of Patients with Maxillofacial Trauma Due to Interpersonal Violence Treated in a Hospital Emergency Room. Dent. Traumatol..

[65] De Macedo Bernardino Í., Santos L.M., Ferreira A.V.P., De Almeida Lima T.L.M., Da Nóbrega L.M., d’Avila S. (2018). Intimate Partner Violence against Women, Circumstances of Aggressions and Oral-Maxillofacial Traumas: A Medical-Legal and Forensic Approach. Leg. Med..

[66] Rodrigues L.G., Barbosa K.G.N., Silva C.J.D.P., Alencar G.P., D’avila S., Ferreira E.F.E., Ferreira R.C. (2020). Trends of Maxillofacial Injuries Resulting from Physical Violence in Brazil. Dent. Traumatol..

[67] Gałczyńska-Rusin M., Pobudek-Radzikowska M., Czajka-Jakubowska A. (2024). Polish Language Adaptation and Validation of the Fonseca Anamnestic Index for Individuals with Temporomandibular Disorders. Dent. Med Probl..

